# The impact of collaborative agglomeration of manufacturing and producer services on carbon emission intensity: Influence mechanism and spatial effect

**DOI:** 10.1371/journal.pone.0295948

**Published:** 2023-12-21

**Authors:** Yanfei Liu, Long Li, Xiaozhuang Yang

**Affiliations:** 1 School of Management, Harbin University of Commerce, Harbin, China; 2 School of Energy and Civil Engineering, Harbin University of Commerce, Harbin, China; China University of Mining and Technology, CHINA

## Abstract

Under the goal of " carbon peaking and carbon neutrality," how to create a low-carbon and green development path for cities is an urgent problem in China. One of the effective ways to solve this problem is through industrial collaborative agglomeration. In this paper, panel data of 276 prefecture-level cities in China from 2010 to 2020 was selected to investigate the relationship between the collaborative agglomeration of manufacturing and producer service and carbon emission intensity by using a fixed-effects model, intermediary model, and SDM in multiple dimensions. The study found that the collaborative agglomeration of manufacturing and producer service industries significantly influences carbon emission intensity with an inverted U-shaped curve, and the agglomeration of manufacturing and producer service industries can influence carbon emission intensity through technological progress. In addition, the inverted U-shaped impact of collaborative agglomeration of manufacturing and producer services on carbon emission intensity has a spatial spillover effect, and the spatial spillover effect generated is stronger than the impact on the local area. Moreover, from the perspective of industry heterogeneity, compared with low-end producer services, the agglomeration of manufacturing and high-end producer services can play a better role in carbon emission reduction. Regarding regional heterogeneity, compared with the central and western regions, the impact of manufacturing and production services agglomeration on carbon intensity is far more obvious in the more economically developed eastern regions.

## 1. Introduction

**Fig 1:**
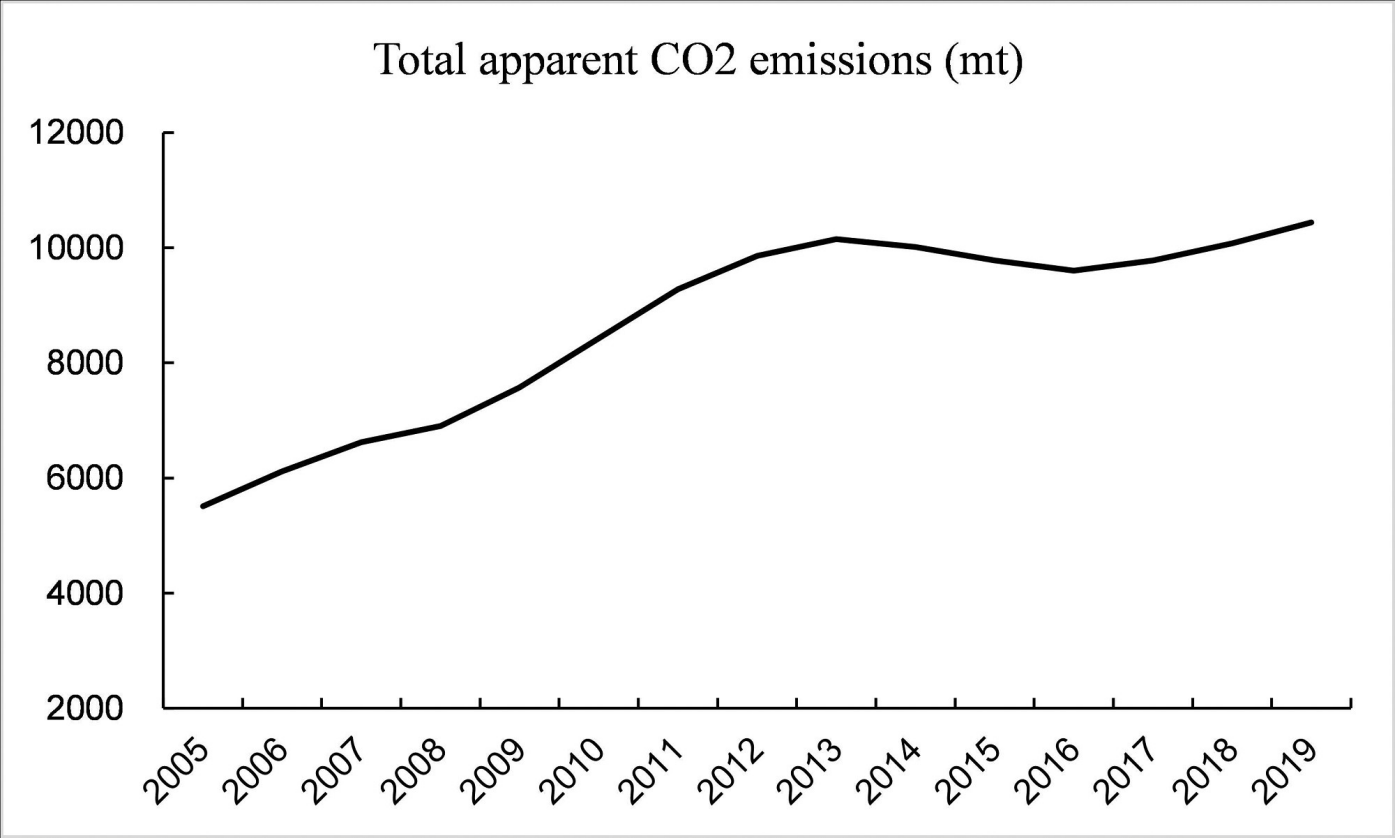
Total apparent carbon dioxide emissions in China from 2005 to 2019.

**Fig 2:**
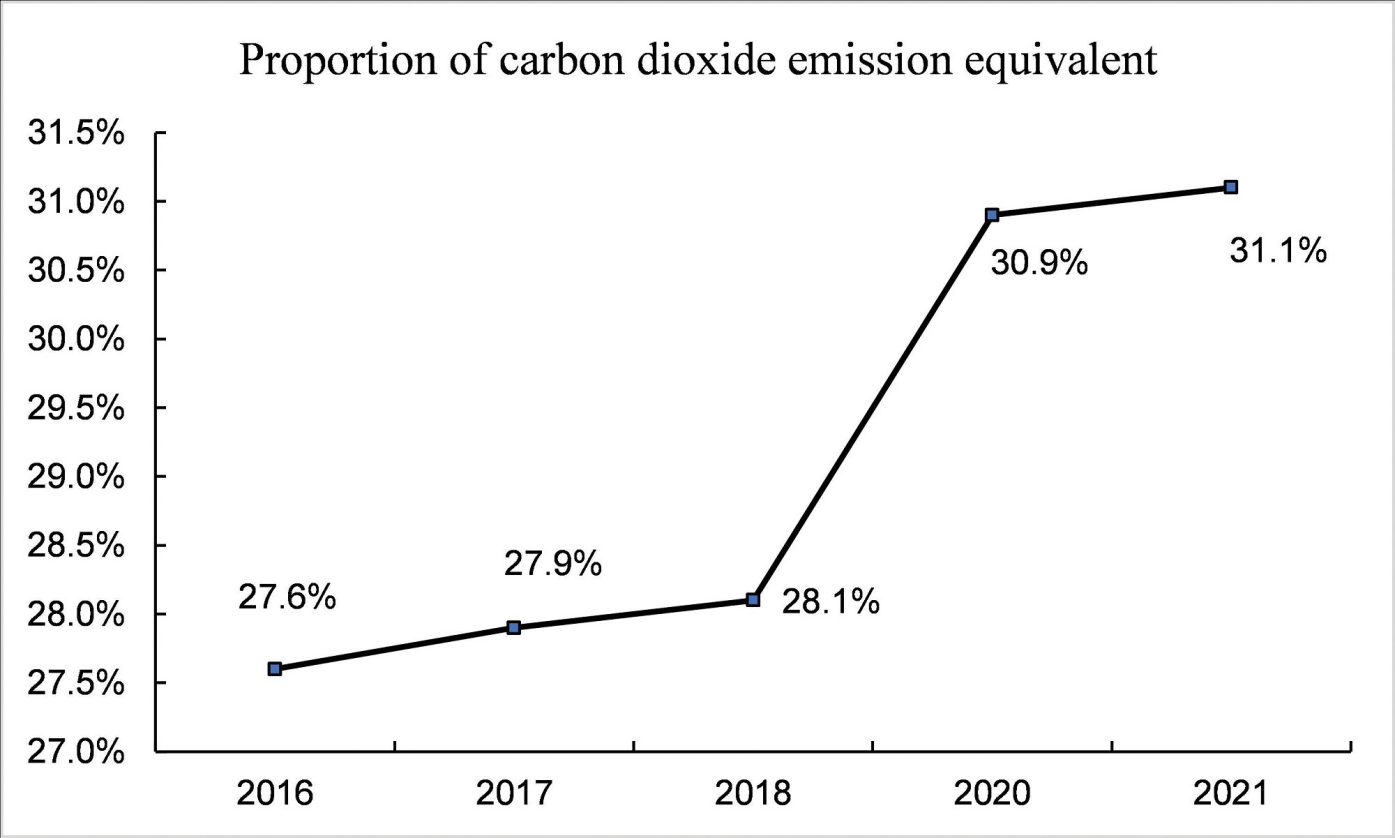
The proportion of China’s carbon dioxide emissions equivalent to global emissions from 2016 to 2021.

**Fig 3:**
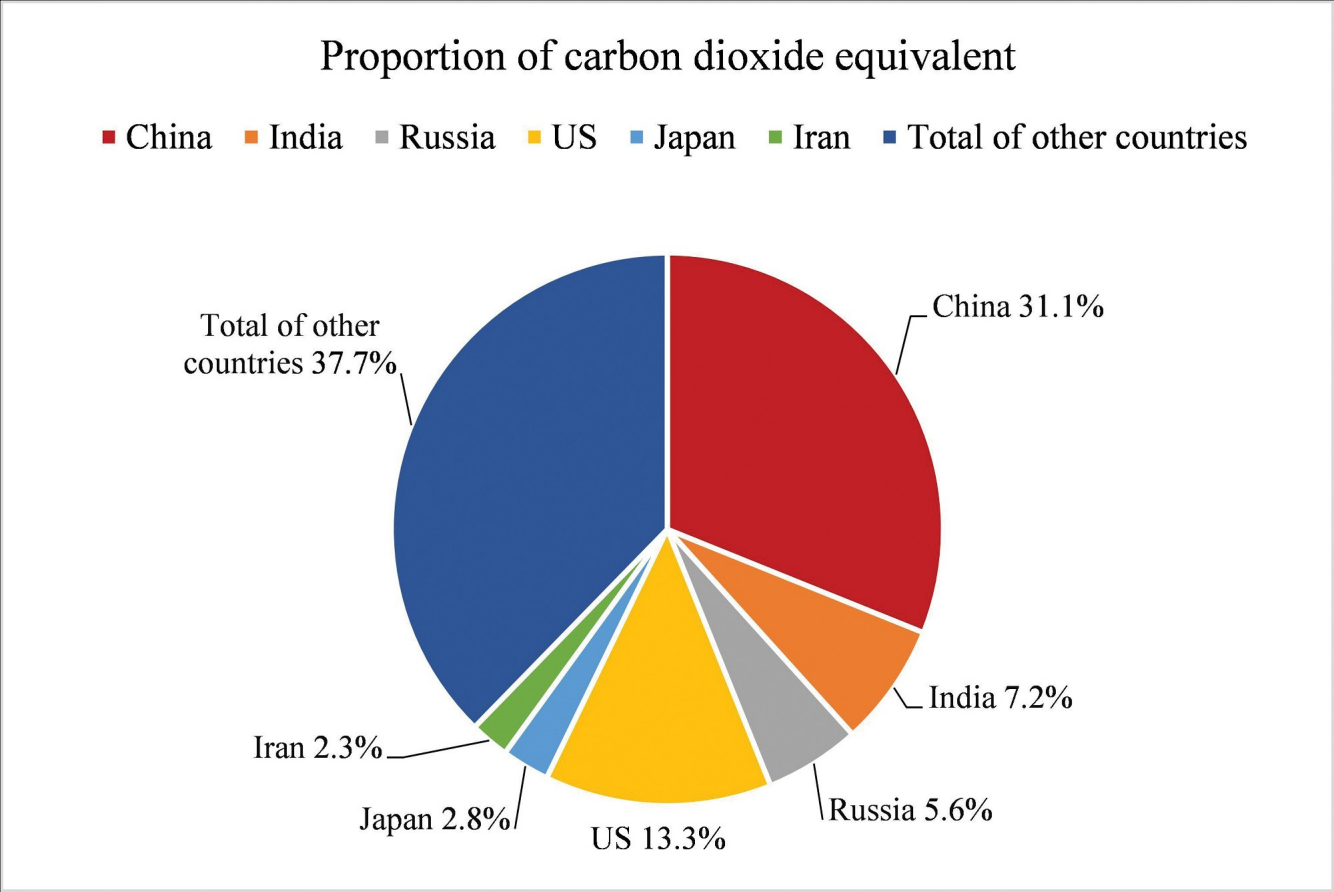
The proportion of global CO2 equivalent emissions by country in 2021.

With the growing problem of global climate change, various countries have taken note of environmental issues and started to advocate a low-carbon economy. In recent years, China’s economy has been developing rapidly. In the process of industrialization and urbanization, a lot of energy consumption and carbon dioxide emissions have been produced. According to CEADs data statistics, China’s apparent carbon dioxide emissions have shown an overall upward trend in recent years (Fig 1). According to the BP Statistical Yearbook of World Energy, China’s proportion of carbon dioxide equivalent emissions among all countries has increased in recent years (Fig 2), reaching 31.1% in 2021 (Fig 3). As the nation with the highest percentage of carbon emissions globally, China faces substantial pressure to conserve energy and mitigate emissions. Consequently, energy conservation and emission reduction have emerged as imperative strategic objectives for China’s sustainable economic development in the new era. As a consequence, the central government of China has implemented a range of measures to strengthen the regulation of carbon emission intensity. During the 75th United Nations General Assembly on September 22, 2020, Xi Jinping emphasized China’s commitment to enhancing its national autonomous contribution to the fight against climate change. He announced that China will implement more robust policies and measures to reduce carbon dioxide emissions effectively. China aims to reach the carbon dioxide emissions peak by 2030 and achieve carbon neutrality by 2060. According to the "2022 Annual Report on China’s Policies and Actions to Address Climate Change" published by the Ministry of Ecology and Environment, China’s carbon dioxide emissions per unit of gross domestic product (GDP) will be reduced by more than 65% by 2030 compared with 2005. China’s "14th Five-Year Plan" also emphasized the important strategic position of green development in economic construction, and low-carbon development has become one of the core strategies of China’s new normal economy. All these demonstrate China’s commitment to promoting green and low-carbon development and its proactive approach towards addressing global climate change. Currently, the economy has entered a new normal of sustainable development of growth rate shift and structural adjustment, and the growth mode has changed from single-wheel drive of manufacturing industry to "double-wheel drive" mode of collaborative agglomeration of productive service industry and manufacturing industry [1]. In this context, it is imperative to study the relationship between industrial collaborative agglomeration and carbon emissions, and this paper focuses on the impact of collaborative agglomeration of manufacturing and producer services on carbon emission intensity. As a high emission of pollution, manufacturing has become a focus of attention. As the main body of the national economy, the high pollution of the manufacturing industry has become a severe shackle of our green development [2]. "Made in China 2025" proposed that China should transform itself from a major manufacturing country to a manufacturing powerhouse. Therefore, the transformation and development of the manufacturing industry and the improvement of the environment have emerged as crucial objectives for achieving low-carbon development in China’s economy. As an industry separated from manufacturing, the producer service industry exhibits notable features such as high knowledge intensity, high-tech content, and significant added value. Moreover, it plays a crucial role in all stages of manufacturing production. Hence, the level of collaborative agglomeration in the above two industries will exhibit a location orientation with the increase of industrial correlation [3], which drives the restructuring of the manufacturing industry. Therefore, given the significant industrial interconnection, it is crucial to investigate the relationship between the collaborative agglomeration of manufacturing and producer services and carbon emission intensity. In this paper, panel data from 276 Chinese cities from 2010 to 2020 were used to establish various econometric models to empirically analyze the influence mechanism, spatial spillover effect, and heterogeneity of the collaborative agglomeration of manufacturing and producer services on carbon emission intensity. The purpose of this study is to offer a reference for comprehending the relationship between industrial collaborative agglomeration and carbon emission intensity, as well as to provide a reference for reducing urban carbon emission intensity.

## 2. Literature review

First of all, extensive research has been conducted both domestically and internationally on the correlation between industrial agglomeration and environmental pollution. These studies have yielded diverse findings, which can be broadly classified into three main views. The first view posits that industrial agglomeration exacerbates pollutant emission and environmental deterioration. From the perspective of industrial agglomeration, Virtanen(1998) [4] and Verhoef et al. (2002) [5] found that industrial agglomeration would causes increased emissions of pollutant gases, thus further damage the environment in the agglomeration area. Ciccon (1996) argued that industrial agglomeration leads to excessive concentration of enterprises, which increases energy consumption and thus leads to increased pollution emissions [6]. Leeuw et al. (2001) and Dong et al. (2020) also draw similar conclusions [7, 8]. At the micro level, Martin et al. (2011) argueed that industrial agglomeration can exacerbate environmental pollution [9]. The second view is that industrial agglomeration can reduce carbon emissions and improve the regional environment. Newman et al. (1989) and Hosoe (2006) argued that industrial agglomeration could reduce carbon emissions and improve the regional environment through knowledge spillover and technological innovation [10, 11]. Han et al. (2018) established a spatial econometric model and found that diversified agglomeration could reduce the carbon emission intensity of the local and surrounding regions through positive externalities [12]. From the perspective of producer services, Puga (1999) believed that industrial agglomeration could promote the development of economies of scale and optimize the structure of factor inputs, thus improving environmental quality [13]. The third view holds that there is a nonlinear relationship between industrial agglomeration and carbon emission intensity. Mao et al. (2022) constructed an extended spatial econometric model based on the STIRPAT model and found that there is a significant "N" type relationship between industrial agglomeration and carbon emissions [14]. Ji et al. (2021) studied the impact of industrial agglomeration on carbon emission from the perspective of urban characteristics and institutional characteristics. They found a significant inverted U-shaped relationship between industrial agglomeration and carbon emission [15].

Secondly, with the advancement of research, scholars have initiated investigations into the phenomenon of industrial diversification agglomeration. Different modes of industrial agglomeration will have different impacts on carbon emissions [16]. Jacobs et al. (2014) believed that industrial collaborative agglomeration has a greater potential to reduce carbon emissions compared to single agglomeration because industrial collaborative agglomeration is easier to generate economies of scale [17]. By establishing a spatial econometric model, Miao et al. (2020) found that a single industrial agglomeration has a positive correlation with carbon emissions and exacerbate environmental pollution. However, the collaborative agglomeration of the secondary and tertiary industries can have an inverted U-shaped impact on carbon emissions due to the agglomeration externality [18]. Hou et al. (2023) conducted a study on 283 prefecture-level cities in China and arrived at a similar conclusion as Miao Jianjun [19]. Wang et al. (2019) focused on industrial environmental pollution and found that diversified agglomeration could significantly reduce carbon dioxide emissions and other polluting gases than simple agglomeration [20]. Studies on the collaborative agglomeration of secondary and tertiary industries have reached a relatively mature. However, a certain degree of controversy exists surrounding the research conducted on the collaborative agglomeration of manufacturing and productive service industries concerning pollution emissions. The two main viewpoints can be classified as follows: One viewpoint argued that the collaborative agglomeration of manufacturing and productive service industries can reduce pollution emissions. Sun and Liu (2021) found that the agglomeration of manufacturing and production service industries can significantly improve carbon emissions efficiency by constructing an econometric model and a fractional-order gray prediction model [21]. From a micro perspective, Bao (2022) found that the collaborative agglomeration of manufacturing and production service industries can enhance firm productivity through learning, cost, and competition effects. Consequently, this leads to a reduction in the pollution emission intensity of firms [22]. Wang and Zhou (2022) conducted an empirical study on the economic development zone, focusing on policy effects and mechanisms. Their research findings indicate that effective coordination between manufacturing and low-end producer services could mitigate the carbon growth effect in the economic development zone [23]. Another viewpoint argued that a non-linear relationship existed between the collaborative agglomeration of manufacturing and productive services and carbon emission intensity. Zhao et al. (2022) and Tang (2023) conducted their research in the Yangtze River Economic Belt. They discovered a relationship characterized by an inverted U-shape between the collaborative agglomeration of manufacturing and producer services and pollution emission [16, 24]. Li and Liu (2022) also reached the same conclusion [25]. Meng and Xu (2022), on the other hand, concluded that there was a significant N-shaped relationship between the collaborative agglomeration of manufacturing and production line services and carbon intensity [26].

Finally, to sum up, the research on industrial agglomeration has achieved considerable results. Among them, there are relatively more studies on the relationship between single industrial agglomeration and pollution emission, while relatively few studies on the relationship between industrial collaborative agglomeration and environmental pollution. Ignoring industrial collaborative agglomeration is not conducive to developing a green and low-carbon economy [19]. Moreover, most of the research objects of industrial collaborative agglomeration have focused on the secondary and tertiary industries, and there are relatively few research objects of collaborative agglomeration with manufacturing and productive service industry agglomeration as the research object of synergistic agglomeration. Furthermore, a limited number of studies have examined the effects of the collaborative agglomeration of manufacturing and producer services on carbon emissions. Finally, in previous literature, the majority of studies have primarily concentrated on the provincial level or a specific economic region, making capturing spatial effects and heterogeneity challenging. Therefore, this paper utilized panel data from 276 cities at or above the prefecture level to analyze the influence mechanism and spatial spillover of collaborative agglomeration between manufacturing and producer services on carbon emission intensity. Additionally, the empirical analysis of industry heterogeneity and regional heterogeneity were carried out. The possible contributions of this paper are outlined as follows: First, based on the city level, this paper studied the impact of the agglomeration of manufacturing and producer services on carbon emission intensity and its spatial spillover effect, which enriches the research on industrial collaborative agglomeration and pollution emission. Second, this paper incorporated industrial collaborative agglomeration, technological progress, and carbon emission intensity into a comprehensive analytical framework to analysis the possible path of carbon emission reduction in the collaborative agglomeration between manufacturing and producer services. Moreover, the study aimed to enhance its comprehensiveness by conducting further analysis on industry heterogeneity and regional heterogeneity.

## 3. Theoretical analysis and research hypothesis

### 3.1 The relationship between collaborative agglomeration of manufacturing and producer services and carbon emission intensity

Industrial agglomeration refers to the occurrence of a cluster of interconnected businesses that exhibit a high level of concentration within a particular geographical region. Marshall pointed out that "externalities" will be generated in the concentration process. Jacobs later highlighted the externalities generated by industrial agglomeration are the division of labor and complementary effects of interrelated firms clustered together. He also pointed out that diversified agglomeration can generate knowledge spillover across various industries, thus promoting enterprise innovation and regional economic development [27]. In addition, this complementary system of labor division has the potential to effectively decrease expenses and conserve energy. It also facilitates the exchange of resources and technologies between firms through the dissemination of knowledge. Therefore, enterprises with forward and backward linkages can exchange technology and knowledge more conveniently and create new, more advanced, "clean" technologies to reduce environmental pollution [16].

As enterprises connected forward and backward, the manufacturing and producer service industries have a strong correlation and supply and demand relationship, so they are very suitable for the research objects of industrial collaborative agglomeration [28]. According to previous relevant research literature, the influence of industrial co-agglomeration on carbon emission intensity can be divided into positive externalities and negative externalities. In the initial phase of industrial collaboration, commonly referred to as the running-in phase, companies share knowledge and technology through the integration of upstream and downstream industry chains. However, the process of knowledge exchange during the initial stage of agglomeration is slow, and there is room for improvement in the market structure during this phase. The rapid expansion of capacity will result in a significant rise in production factors, leading to an increase in carbon emissions, and the positive externalities of industrial collaborative agglomeration cannot play a role. When the synergistic clustering of manufacturing and productive services reaches the mature stage, the running-in period of agglomeration has passed, and the agglomeration process between the two industries is more comprehensive and smooth. The production service industry plays a crucial role in facilitating the provision of intermediate goods required by the manufacturing industry. Additionally, a portion of the manufacturing output can be utilized by producer services to enhance their service industrialization process, thereby enhancing overall production efficiency. Therefore, the delicate and circular production process formed by the collaborative agglomeration of manufacturing and producer services can save resources and energy and reduce carbon emission intensity [29]. The positive externality of industrial collaborative agglomeration plays a role. Therefore, this paper proposes Hypothesis 1:

Hypothesis 1: There is a significant inverted U-shaped relationship between the collaborative agglomeration of manufacturing and producer services and carbon emission intensity. In improving the level of industrial collaborative agglomeration, carbon emission intensity will be promoted first and then suppressed after reaching a certain level.

### 3.2 Mechanism analysis

Studies have shown that industrial collaborative agglomeration can impact technological progress [30–32], and technological progress is a meaningful way to curb the intensity of carbon emissions [33]. Therefore, this paper argued that the collaborative agglomeration of manufacturing and producer services can influence carbon emissions through technological progress. The specific analysis is as follows:

As backward and forward industries, the division of labor between manufacturing and productive service industries can share production technology and resources in agglomeration, strengthening the rational allocation of resources. Thus, the reduction in costs of innovation and the enhancement of technological progress can be achieved [31]. When mature communication mechanisms are gradually established within the two industrial clusters, enterprises will unconsciously accept unified standards, thus reducing risks in cooperation. Moreover, establishing stable cooperation between firms can significantly increase their motivation to pursue long-term profits, thus promoting technological innovation [34]. Venable believed that fierce competition will be generated in the collaborative agglomeration of manufacturing and producer services, thus weeding out low-technology and inefficient enterprises. It can also influence the level of technological progress in the region from the side [35]. In addition, if the collaborative path of two major industries within a certain agglomeration area forms a fixed pattern, it may also exert a detrimental influence on technological progress [28].

The above theory suggests that synergistic industrial agglomeration influenced technological progress, and technological progress can also impact carbon emission intensity. On one hand, technological progress can gradually realize automated production and reduce the dependence on labor, thus promoting the transformation of labor-intensive industries to technology-intensive industries. It, therefore, can improve energy use efficiency and reduce carbon emission intensity [36]. On the other hand, technological progress can not only create new green technologies that provide cleaner production processes, but also integrate and utilize existing green technologies. It can improve technical efficiency and reduce carbon intensity.

In summary, technological progress plays a mediating role in the process of collaborative industrial agglomeration affecting carbon emission intensity. Thus, hypothesis 2 is formulated:

Hypothesis 2: The collaborative agglomeration of manufacturing and producer services can affect carbon emission intensity through technological progress.

### 3.3 Spatial spillover effect

Industrial collaborative agglomeration facilitates the sharing of resources and production factors, and the production technology in resource sharing can spread rapidly through the industrial collaboration within the agglomeration region, thus causing the interregional carbon emission correlation [37, 38]. At the initial stage of industrial collaborative agglomeration, the collaborative agglomeration of manufacturing and producer services within a region can impact the carbon emission intensity of the region and its surrounding areas through negative externalities. In the initial agglomeration phase, there is a concentration of various production factors, resulting in a higher carbon dioxide emission. However, carbon dioxide is diffusive and fluid, which indirectly affects the carbon emission intensity of neighboring areas. In addition, the collaborative agglomeration of manufacturing and producer services will exert a siphoning influence on the higher-level production factors in the neighboring regions during the initial stage, forcing out some enterprises with high pollution emissions to the surrounding areas [39], thus increasing the carbon emission intensity of the surrounding areas. In the mature stage of industrial collaborative agglomeration, the synergistic effect of the agglomeration area tends to be stable, and the knowledge spillover between enterprises can have an impact on the surrounding area [7], which can improve the production technology of the surrounding area, save energy, and reduce environmental pollution. In addition, the existence of a "demonstration impact" between firms in neighboring areas [38], and the sense of competition between enterprises will enable them to learn from each other. When enterprises in this region develop rapidly through agglomeration to improve production technology and produce more "green" products, enterprises in neighboring areas will also learn this development mode to reduce pollution emissions. In summary, the initial phase of collaborative agglomeration among manufacturing and production service industries will promote the carbon emissions in nearby regions. In contrast, the mature stage of collaborative agglomeration will suppress neighboring regions’ carbon emission. Thus, hypothesis 3 is formulated:

Hypothesis 3: The collaborative agglomeration of manufacturing and producer services can have an inverted U-shaped influence on the carbon emission intensity of neighboring areas through the spatial spillover effect.

## 4. Materials and methods

### 4.1 Model

#### 4.1.1 Fixed effects model

In order to test the above hypothesis, this paper first constructed a fixed effect model to empirically analyze the impact of the industrial synergy agglomeration on carbon emission intensity, as shown in Eq (1):

$${\mathrm{CEI}}_{it}=\alpha_{0}+\alpha_{1}{\mathrm{COA}}_{it}+\alpha_{2}{{COA}^{2}}_{it}+\alpha_{c}X_{it}+\mu_{i}+\sigma_{t}+\varepsilon_{it}$$ (1)

In Formula (1), i represents the city and t represents the year; CEI_*it*_ represents the carbon dioxide emission intensity of city i in year t; COA_*it*_ represent the index of collaborative agglomeration of manufacturing and producer services, *COA*^2^_*it*_ represent the square term of the index of collaborative agglomeration of manufacturing and producer services; X is a series of control variables; *α*_0_ represents the intercept term, and *α*_1_、*α*_2_、*α*_c_ are the estimated coefficients of the variables. *μ*_*i*_ is the individual effect, *σ*_*t*_ is the time effect, and *ε*_*it*_ is the random disturbance term.

In addition, in order to further analyze whether the collaborative agglomeration of manufacturing and producer service industries affects the carbon emission intensity by influencing technological progress, this paper uses the mediation model to verify it by two methods: one is to refer to Wen et al. [40] and use the stepwise regression method to verify it; the other is to use the Bootstrap method with higher statistical validity according to Hayes [41]method test. The econometric model is as follows:

$${\mathrm{TE}}_{it}=\beta_{0}+\beta_{1}{\mathrm{COA}}_{it}+\beta_{2}{{COA}^{2}}_{it}+\beta_{c}X_{it}+\mu_{i}+\sigma_{t}+\varepsilon_{it}$$ (2)

$${\mathrm{CEI}}_{it}=\gamma_{0}+\gamma_{1}{\mathrm{COA}}_{it}+\gamma_{2}{{COA}^{2}}_{it}+\gamma_{3}{\mathrm{TE}}_{it}+\gamma_{c}X_{it}+\mu_{i}+\sigma_{t}+\varepsilon_{it}$$ (3)

In this formula, TE is the mediating variable: technological progress, and other indicators are the same as above. Formula (2) is the regression model of the cooperative agglomeration of manufacturing and producer services on technological progress. Formula (3) is based on Formula (1) with the addition of technological progress as a intermediary variable. In this paper, we first determine whether the mediating effect exists according to the significance of the stepwise regression method and then verify whether the mediating effect exists again according to whether the 95% confidence interval contains 0 by the Bootstrap method.

#### 4.1.2 Spatial econometric model

Previous studies have shown that there is a spatial correlation between carbon emission intensity [42]. Over time, the barriers between regions are decreasing and factors and products are moving more freely between neighboring cities, which makes the action scale of spatial spillover effects between regions gradually shrink. Furthermore, numerous studies in economic geography have consistently found that cities play a central role in regional development and serve as the primary contributors to carbon emissions. Carbon dioxide and other gas emissions affect the local ecological environment and spill over to neighboring regions, exhibiting a significant correlation in space. Based on the aforementioned context, this paper finally used the spatial econometric model to study the spatial impact of industrial collaborative agglomeration on carbon emission intensity. The specific model is as follows:

$${CEI}_{it}=\alpha_{0}+\rho \sum_{j=1}^{N}W_{ij}{CEI}_{it}\;+\alpha_{1}{\mathrm{COA}}_{it}+\varphi_{1}\sum_{j=1}^{N}W_{ij}{COA}_{it}\;+\alpha_{2}{{COA}^{2}}_{it}+\varphi_{2}\sum_{j=1}^{N}\;W_{ij}{{COA}^{2}}_{it}+\alpha_{c}X_{it}+\varphi_{c}\sum_{j=1}^{N}\;W_{ij}X_{it}+\mu_{i}+\sigma_{t}+\varepsilon_{it}$$ (4)

In this formula, ρ is the spatial autoregressive coefficient; *φ*_1_、*φ*_2_ and *φ*_*c*_ are the estimation coefficients of each variable and space interaction terms. *ε*_*it*_ is the spatial error autocorrelation. *W*_*ij*_ is the space weight matrix. In this paper, the 0–1 spatial weight matrix is used for conventional estimation and the spatial inverse distance weight matrix is used for robustness testing, defined as follows:

Definition of 0–1 space weight matrix with Queen adjacency rule:

$$W_{ij}=\{\begin{array}{l} 0\;\mathrm{Area}\;"i"\;\mathrm{and}\;"j"\;\mathrm{are}\;\mathrm{not}\;\mathrm{adjacent}.\\ 1\;\mathrm{Area}\;"i"\;\mathrm{and}\;"j"\;\mathrm{are}\;\mathrm{adjacent}. \end{array}$$ (5)

Definition of space inverse distance weight matrix:

$$W_{ij}=\frac{1}{d_{ij}}$$ (6)

Where, *d*_*ij*_ is the geographical distance between the central coordinates of region i and region j.

### 4.2 Variables selection and data sources

#### 4.2.1 Variables selection

Dependent variable: carbon dioxide emission intensity (CEI). This paper adopted the ratio of total carbon dioxide emission intensity to GDP to represent urban carbon emission intensity [43]. The current measurement of carbon emissions is mainly calculated using the sectoral method and the apparent emission accounting method. Due to the availability of city-level data, this paper draw on the practice of Shan et al. [44] and used carbon emissions data generated by easily accessible fossil fuels to represent the total carbon emissions of cities. Specifically, the apparent consumption of raw coal, crude oil, and natural gas is multiplied by the corresponding carbon conversion factor while excluding the use of non-energy apparent consumption.

*Independent variable*. Collaborative agglomeration of manufacturing and producer services (COA). There are three methods to measure industrial collaborative agglomeration: the E-G index method, the relative difference index method, and the relative index method integrated with location entropy. Previous studies mostly used the relative index method integrated with location entropy, which can reflect both the quality and depth of collaborative agglomeration. Therefore, this paper draw on the thoughts presented by Ellison et al. [45] and the method presented by Zhang et al. (2017) [46] to calculate the collaborative agglomeration index of manufacturing and producer services. The specific formula is as follows:

$${Coa}_{ms}=(1-\frac{\left|LQ_{m}-LQ_{S}\right|}{LQ_{m}+LQ_{S}})+|LQ_{m}+LQ_{S}|$$ (7)

$$LQ_{ij}=\frac{\frac{q_{ij}}{q_{j}}}{\frac{q_{i}}{q}},i=m,s$$ (8)

In Formula (7), *Coa*_*ms*_ *i*s the co-agglomeration index of manufacturing industry and producer services, where m indicates manufacturing industry and s indicates producer services; *LQ*_m_ represents the cluster index of manufacturing, and *LQ*_*S*_ represents the cluster index of producer services. *LQ*_m_ and *LQ*_*S*_ are constructed by location entropy index, and the calculation method is shown in Formula (8). *LQ*_*ij*_ means the location quotient index of i(i = m,s) industry in j city, *q*_*ij*_ is the number of employment in i industry in j city, *q*_*j*_ is the number of employment in manufacturing and producer services in j city, *q*_*i*_ is the number of employment in i industry in the whole country, q is the total number of employment in manufacturing and producer services in China.

*Mediating variable*: *Technological progress (TP)*. This paper used the number of regional patent applications as the representation index of technological progress. Considering that there is an unavoidable lag between patent applications and patent grants, the number of patent applications can objectively reflect the level of technological progress more than the number of patent grants [47].

*Control variables*. In order to control other factors that have an impact on carbon emission intensity, the following control variables were selected in this paper concerning existing relevant studies: (1) energy intensity (EI), expressed by total energy consumption. Energy intensity is an important cause of environmental pollution [48, 49]. In the process of burning coal and other fossil fuels, sulfur dioxide and carbon dioxide are released. (2) Economic development level (PGDP), represented by per capita GDP, an economic development level will have a significant impact on the carbon emission intensity of a region [50]. (3) Degree of openness (FD), which is measured by the ratio of FDI to GDP, may introduce new products and technologies that make enterprises’ production activities more "green", thus reducing carbon dioxide emissions. It may also deteriorate the environment of the introduced country, thus causing the "pollution refuge" effect [51]. (4) Urbanization rate (UR) is expressed by the ratio of the permanent urban population to the total permanent urban population. With the acceleration of the urbanization process, the population gathers in cities, which may increase the demand for energy consumption [52]. (5) Environmental regulation (ER). Based on the study of Zhang and Chen (2021), this paper measured environmental regulation by calculating the ratio of the word frequency of environmental vocabulary to the word frequency of work report from prefecture-level city government [53]. (6) Human capital (HC), which is expressed as the ratio of the number of general undergraduates and above population to the city’s resident population. Human capital may reduce carbon emissions by enhancing firms’ efficiency through improved technological proficiency and innovation, ultimately leading to a decrease in carbon emissions. It is also possible that a large number of laborers will be concentrated in pollution-intensive industries and confined to the lower end of the manufacturing value chain, which is not conducive to the promotion of cleaner production technologies [16]. This ultimately leads to a slight increase in carbon emission intensity.

#### 4.2.2 Data sources

Considering the availability and completeness of data, this paper selected data from 276 prefecture-level cities in China from 2010 to 2020 as the research object. Carbon emission and energy-related data were obtained from the China City Statistical Yearbook, China Energy Statistical Yearbook, and the statistical yearbooks of each prefecture-level city; Environmental regulation data come from the work reports of local governments; The industrial collaborative agglomeration-related variables and the rest of the control variables were obtained from the China Urban Statistical Yearbook. The missing values were filled by linear interpolation, and the dataset was subjected to logarithmic transformation to mitigate the impact of dimensionality and heteroskedasticity. The descriptive statistics of all variables are shown in Table 1 below:

**Table 1:** Descriptive statistics.

variable	symbol	description	Observations	Mean	min	max
Carbon emission intensity	CEI	*CO*_2_ emission intensity /GDP	3036	0.390	0.023	3.385
Collaborative agglomeration of manufacturing and producer services	COA	Index of Collaborative Agglomeration of manufacturing and producer services	3036	2.445	0.183	4.430
Energy intensity	EI	Total energy consumption	3036	4.493	0.129	8.392
Economic development level	PGDP	Logarithm of GDP per capita	3036	10.688	8.576	13.056
Degree of openness	FD	FDI/GDP, Logarithmic processing	3036	4.477	0.098	7.052
Urbanization rate	UR	Urban resident population / City resident population	3036	0.548	0.197	1
Environmental regulation	ER	The word frequency ratio of environmental vocabulary was processed logarithmically	3036	3.475	0	4.827
Human capital	HC	The ratio of the number of general undergraduate and above population to the city’s resident population.	3036	0.018	0.000	0.128
Technological progress	TP	Logarithm of the number of patent applications	3036	7.308	2.197	12.312

## 5. Empirical analysis

### 5.1 Effects of collaborative agglomeration of manufacturing and producer services on carbon emission intensity under non-spatial panel model

#### 5.1.1 Baseline regression analysis

This paper mainly studied the impact of collaborative agglomeration of manufacturing and producer services on carbon emission intensity and used stata15.1 software to conduct an empirical study. This paper first incorporated the linear term of collaborative agglomeration of manufacturing and producer services into the regression model. The results are shown in column (1) of Table 2, which shows that the coefficient of the collaborative agglomeration of manufacturing and producer services is positive but not significant when other variables are controlled. It indicates that there is a certain linear relationship between the collaborative agglomeration of manufacturing and producer services on carbon emission intensity. However, this relationship has not to be verified in the statistical model. Therefore, next, we included the square term of the collaborative agglomeration of manufacturing and producer services into the model, and the results are shown in column (2) of Table 2. The coefficient of the linear term of the collaborative agglomeration of manufacturing and producer services is 0186 and significant at the 1% level. The square term coefficient of collaborative agglomeration of manufacturing and producer services is -0.039 and is significant at 1% level. It indicates that there is a significant inverted-U curve relationship between collaborative agglomeration of manufacturing and producer services and carbon emission intensity, which indicates that hypothesis 1 is valid. Specifically, the inflection point at which the collaborative agglomeration of manufacturing and producer service industries inhibits carbon emissions intensity is 2.3996. When the collaborative agglomeration index is lower than 2.3996, the collaborative agglomeration of manufacturing and producer services is in the running-in stage, and the rapid expansion of production capacity leads to the increase of the agglomeration of production factors, increasing carbon emission intensity. When the collaborative agglomeration index of manufacturing and productive service industries exceeds 2.3996, the running-in period is over, and the collaborative process between the two industries establishes a virtuous cycle of production process between enterprises through knowledge spillover and technology exchange, which can save energy and suppress carbon emission intensity. Moreover, from the results, overall, the average index of collaborative agglomeration between manufacturing and productive service industries in China is 2.545, which has exceeded the inflection point and exerted its positive externality to suppress carbon emission intensity.

**Table 2:** Results of baseline regression.

	(1)	(2)
	CEI	CEI
COA	0.005 (0.015)	0.186*** (0.067)
COA^2^		-0.039*** (0.014)
EI	0.251*** (0.015)	0.250*** (0.015)
PGDP	-0.312*** (0.023)	-0.313*** (0.022)
FD	-0.008*(0.004)	-0.009** (0.004)
UR	0.427*** (0.105)	0.424*** (0.105)
ER	0.011*(0.007)	0.010 (0.006)
HC	1.614* (0.887)	1.674*(0.891)
_cons	2.433*** (0.208)	2.265*** (0.213)
N	3036.000	3036.000
*R* ^2^	0.902	0.902

Note: In brackets are the corresponding robust standard errors

* means significant at 10% level

** means significant at 5% level

*** means significant at 1% level, the same below.

Among the control variables, the coefficient of energy intensity is 0.250 and is significant at 1% level. Every 1 unit increase in energy intensity will increase carbon emission intensity by 0.250 units. It can be inferred that an increase in energy intensity will result in an increase in carbon emission intensity. It is because energy consumption is the primary source of pollution emissions; coal and other fossil fuels in the burning process will release sulfur dioxide and carbon dioxide. This result is in accordance with the established fact. The coefficient of GDP per capita is negative and significant at the 1% level. It shows that enhancing the economic level can effectively mitigate the intensity of carbon emissions. This may be because, in areas with better economic development, companies can adopt more "clean" technologies, reducing the emission of pollutants. The coefficient of the degree of openness is negative and statistically significant relationship at the 5% level, suggesting that the degree of openness can significantly inhibit carbon emission intensity. It is because the degree of openness can serve as a catalyst for attracting high-tech enterprises, whose production activities are more "green," thereby contributing to the reduction of carbon emission intensity [37]. The coefficient of the urbanization rate is significantly positive at the level of 1%. With the continuous acceleration of urbanization, there is a convergence of rural population towards cities, resulting in an expansion of the population scale and increased infrastructure. This phenomenon leads to a higher consumption of ecological resources, consequently increasing the intensity of carbon emissions. The coefficient of environmental regulation is positive but insignificant, indicating that the implementation of relevant environmental policies is not ideal. The coefficient of human capital is positive at the level of 10%, which is in accordance with the findings of Zhao et al [16]. China is still a global manufacturing factory, and many labor forces may be concentrated in pollution-intensive industries, which is not beneficial to reducing carbon emission intensity.

#### 5.1.2 Analysis of intermediary mechanism

The baseline regression results in Table 2 show that there is a significant inverted-U curve relationship between the collaborative agglomeration of manufacturing and producer services and carbon emission intensity. Is technological progress an essential mechanism for the collaborative agglomeration of manufacturing and producer services to affect carbon emission intensity? Therefore, this paper used the mediation model mentioned above to test. Firstly, regression is performed on the model (2). Column (1) in Table 3 shows the regression results of the collaborative agglomeration of manufacturing and producer services on technological progress. The coefficients of the linear term and the square term of the collaborative agglomeration of manufacturing and producer services are significantly negative and positive, indicating that there is a U-shaped curve effect of collaborative agglomeration of manufacturing and producer services on technological progress, which is first inhibited and then promoted. Then, model (3) is tested by including the collaborative agglomeration of manufacturing and producer services and technological progress together in the regression model. The results are shown in column (2) of Table 3. There is still a significant inverted-U curve relationship between the collaborative agglomeration of manufacturing and producer services on carbon emission intensity, and the coefficient of technological progress is negative and significant at the 5% level. We combine the regression results of model (1) and model (2) to show that technological progress plays the role of incomplete intermediaries. In other words, the collaborative agglomeration of manufacturing and producer services will indirectly suppress carbon emission intensity through technological progress. Hypothesis 2 holds. Moreover, the coefficients of both the linear term and the square term of the collaborative agglomeration of manufacturing and producer services decreased after adding the technological progress of the intermediate variable. In addition, the coefficient of technological progress is -0.022 and is significant at 5% level, indicating that regional technological progress can significantly reduce carbon emission intensity.

**Table 3:** Test results of intermediate effect of stepwise regression method.

	(1)	(2)
	TP	CEI
COA	-1.697*** (0.568)	0.149** (0.071)
COA^2^	0.319*** (0.111)	-0.032** (0.015)
TP		-0.022** (0.011)
EI	0.079** (0.033)	0.251*** (0.015)
PGDP	0.454*** (0.066)	-0.303*** (0.023)
FD	0.012 (0.013)	-0.009** (0.004)
UR	1.098*** (0.260)	0.447*** (0.108)
ER	0.014 (0.024)	0.010 (0.006)
HC	2.579 (1.953)	1.729* (0.889)
_cons	1.326 (0.840)	2.294*** (0.208)
N	3036.000	3036.000
*R* ^2^	0.942	0.903

For further analysis, in model (1), without the addition of technological progress as a mediating variable, the inflection point at which the collaborative agglomeration of manufacturing and producer services is 2.3996. It means that when the collaborative agglomeration index exceeds 2.3996, collaborative agglomeration of manufacturing and producer services will have a suppressive effect on carbon emission intensity. In the total of 3036 samples, it was observed that 1676 samples exceeded the inflection point and exerted the inhibition effect of collaborative agglomeration between manufacturing and producer services on carbon emission intensity. Currently, the average value of the collaborative agglomeration index for manufacturing and producer services is 2.4446, which exceeds the inflection point. It indicated that the collaborative agglomeration level of manufacturing and producer services in China is generally distributed on the right side of the inverted U-shaped curve, showing a suppressing effect on carbon emission intensity. In model (3), when technological progress is added as the intermediary variable, the inflection point value of the inverted U-shaped curve becomes 2.3443. Compared with the result of the model (1) above, the inflection point value decreases, and the number of samples exceeding the inflection point increases to 1798. It indicates that with the improvement of technological progress, collaborative agglomeration of manufacturing and producer services will exert an inhibitory effect on carbon emission intensity in advance. Hypothesis 2 has been further verified.

In order to test the mediating effect of technological progress again, the Bootstrap method was applied in this paper. The Table 4 shown that the 95% confidence intervals of both direct and indirect effects of technological progress did not contain 0, indicating that technological progress played a mediating role. It means that technological progress is an essential mechanism for the collaborative agglomeration of manufacturing and producer services to suppress carbon emission intensity. Hypothesis 2 is valid.

**Table 4:** Test results of Bootstrap mediation effect.

Intermediate variable	Effect type	Value	Standard error	95% confidence intervals
technological progress	Indirect effect	-.0069	0.0044	[-0.0185, -0.0013]
	Direct effect	-.0318	0.0153	[-0.0669, -0.0054]

### 5.2 Spatial effects of collaborative agglomeration of manufacturing and producer services on carbon emission intensity

#### 5.2.1 Spatial autocorrelation test

**Fig 4:**
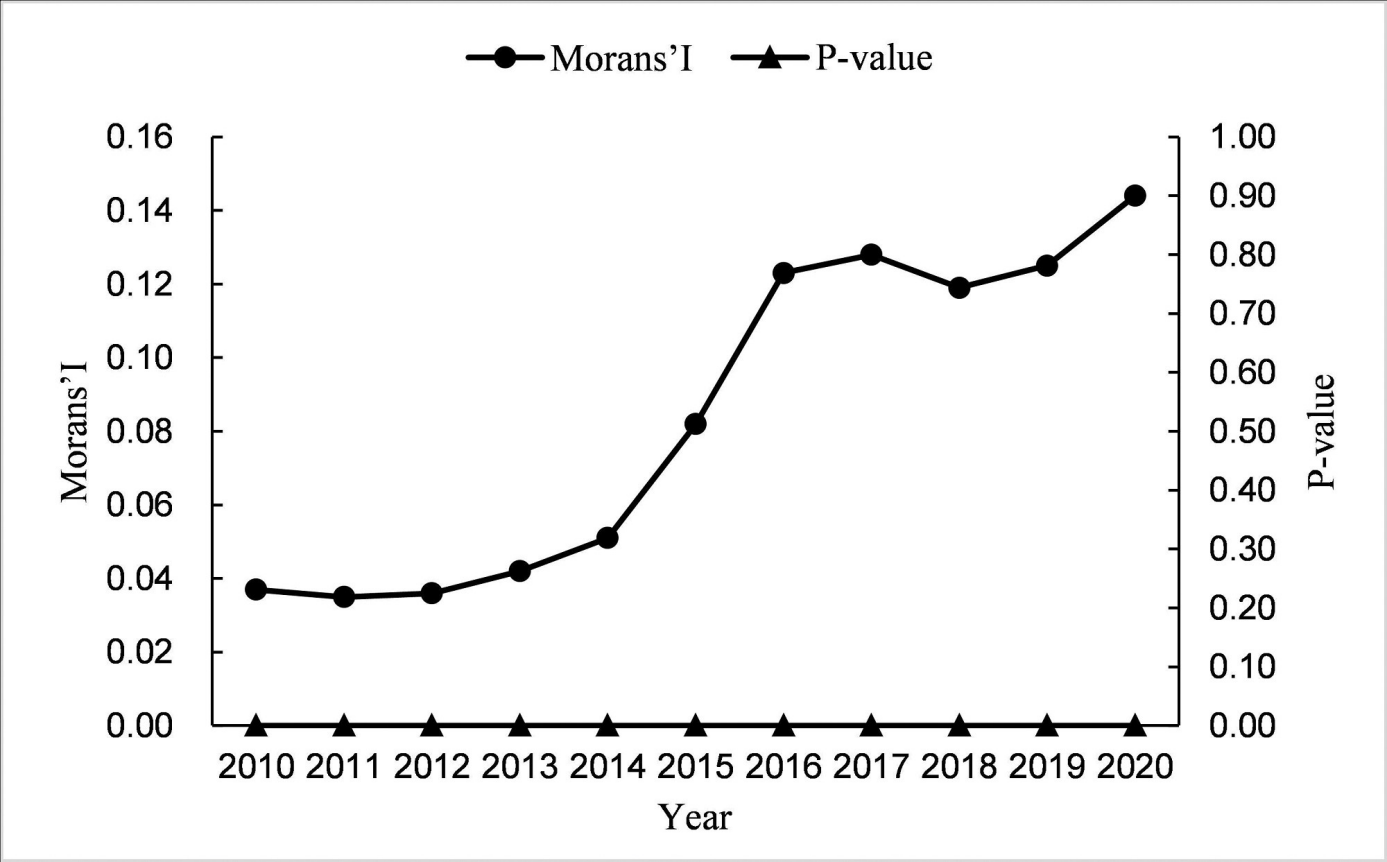
Global Morans’I index and significance level of carbon emission intensity in China.

In order to test the spatial spillover effect of the collaborative agglomeration of manufacturing and producer services on carbon emission intensity, firstly, we have to test the spatial autocorrelation of carbon emission intensity. The global Morans’I index is used in this paper for the test. The results are shown in Fig 4, and the global Morans’I index for carbon emission intensity is positive, and both are significant at the 1% level. This suggests a strong positive spatial correlation between carbon emission intensities.

**Fig 5:**
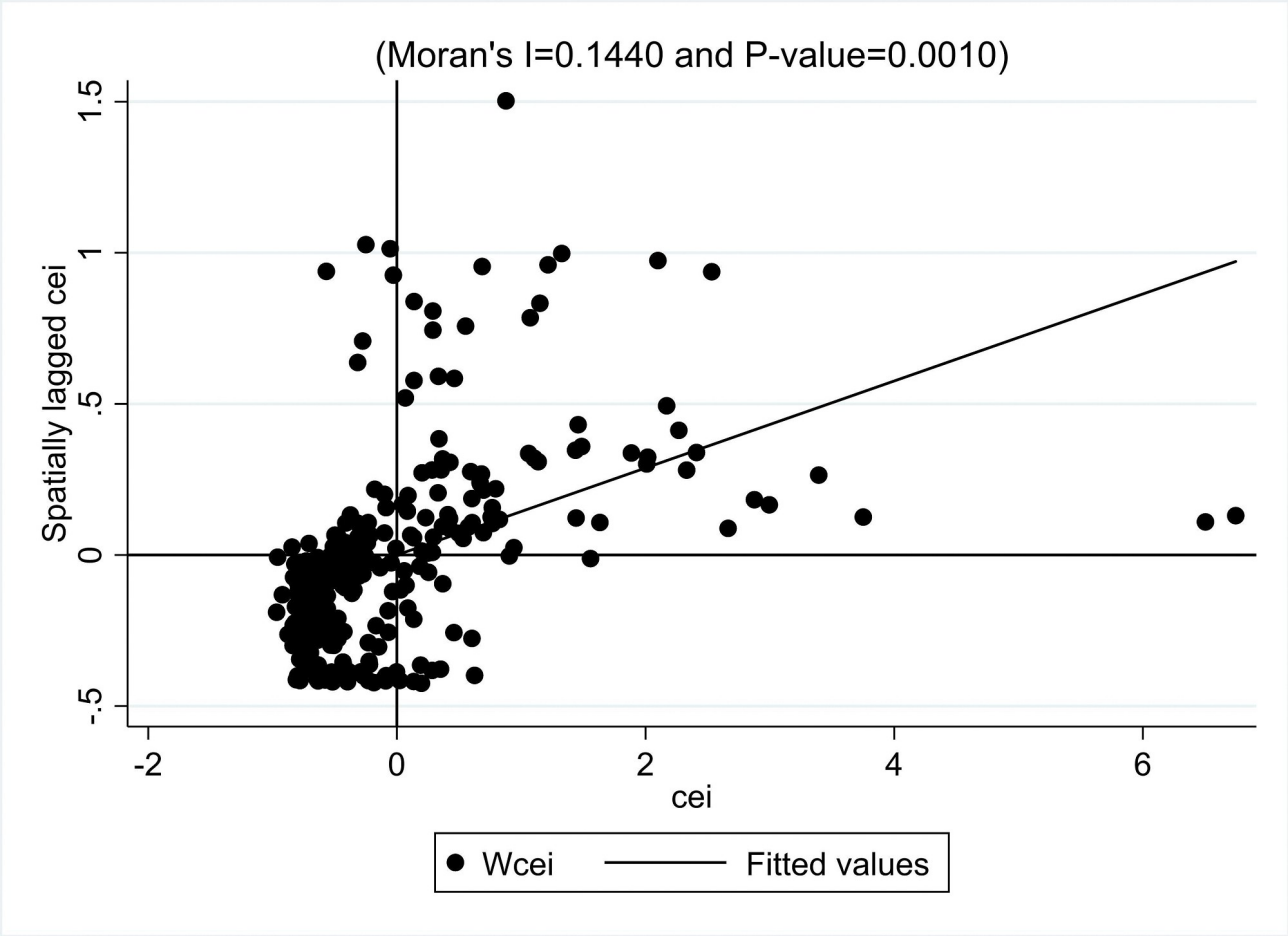
Moran scatter plot of carbon emission intensity in 2020 in China.

Secondly, the Moran scatter plot is used to examine the spatial clustering pattern of carbon emission intensity. This paper reports the sample for 2020. Fig 5 shows that the local Moreland index of China’s carbon emission intensity is mainly distributed in the first and third quadrants, forming an apparent agglomeration trend of "high-high" and "low-low." It indicates that there are primarily positive spatial correlations among cities, and the regions with relatively high (low) carbon emission intensity are surrounded by regions with the same high (low) carbon emission intensity. This is the same as the result of the global Moran index test. Therefore, there is a strong spatial correlation between carbon emission intensity in China’s cities, and it is necessary to use spatial econometric models for empirical verification.

#### 5.2.2 Selection of spatial econometric models

In order to select the appropriate spatial econometric model, the LM test, Hausman test, LR test, and Wald test were conducted in this paper, according to Elhorst [54]. As can be seen from Table 5: Firstly, all of LM passed the significance test. Secondly, the results of the Hausman test are significant at the 1% level, indicating that this paper should choose a fixed-effects model, and taking into account the time factor and individual differences, this paper uses two-way fixed effects. Then, the results of LR tests are significant at the 1% level, indicating that the spatial Durbin model (SDM) is better than the spatial lag model (SAR) and spatial error model (SEM). Finally, the results of the Wald test are also significant at the 1% level, indicating once again that the spatial Durbin model (SDM) is the most appropriate model. In summary, the spatial Durbin model with two-way fixed effects is chosen for the empirical analysis in this paper.

**Table 5:** Test results for the selection of spatial econometric models.

Type of test	Statistics	P-value
LM-error	66.494	0.000
Robust LM- error	3.380	0.066
LM-lag	66.657	0.000
Robust LM-lag	5.544	0.019
Hausman	58.14	0.000
LR-SDM-SAR	53.77	0.000
LR-SDM-SEM	61.94	0.000
Wald-test	54.04	0.000
Wald-testnl	61.56	0.000

#### 5.2.3 Empirical analysis of spatial effects

The test results of SDM are shown in Table 6. The value of ρ in the table is positive and significant at the level of 5%, which indicates that there is a strong spatial dependence on carbon emission intensity. This implies that an increase in carbon emission intensity within a local region will lead to a corresponding increase in carbon emission intensity in the surrounding region. As shown in column (1) of Table 6, the coefficient of the linear term of the collaborative agglomeration of manufacturing and producer service industries is positive, and the coefficient of the square term is negative. Both of them are significant at the 1% level. It indicates that there is a significant inverted U-shaped effect of collaborative agglomeration of manufacturing and producer services on carbon emission intensity, which is consistent with the previous results. Column (2) of Table 6 shows the regression results of the spatial lag term. It can be seen that the coefficient of the linear term of the collaborative agglomeration of manufacturing and producer service industries is positive, and the coefficient of the square term is negative. Both of them are significant at the level of 1%. It suggests that the collaborative agglomeration of manufacturing and producer services in the region also has a significant inverted U-shaped effect on the carbon emission intensity of neighboring regions.

**Table 6:** Regression results of SDM.

Variables	(1)	(2)	(3)	(4)	(5)
	Main	Wx	Direct effects	Indirect effects	Total effects
COA	0.173***	1.660***	0.181***	2.534***	2.715***
	(0.043)	(0.590)	(0.045)	(0.925)	(0.932)
COA^2^	-0.037***	-0.404***	-0.039***	-0.611***	-0.650***
	(0.009)	(0.130)	(0.009)	(0.210)	(0.211)
EI	0.252***	0.157	0.253***	0.334**	0.587***
	(0.007)	(0.098)	(0.006)	(0.159)	(0.161)
PGDP	-0.273***	0.022	-0.274***	-0.071	-0.345***
	(0.015)	(0.071)	(0.015)	(0.084)	(0.082)
FD	-0.009***	-0.015	-0.009***	-0.028	-0.037
	(0.003)	(0.016)	(0.002)	(0.023)	(0.023)
UR	0.422***	-0.660***	0.424***	-0.754**	-0.330
	(0.064)	(0.241)	(0.064)	(0.377)	(0.375)
ER	0.005	0.127**	0.006	0.179**	0.185**
	(0.006)	(0.053)	(0.006)	(0.085)	(0.085)
HC	1.830***	-16.659***	1.750***	-23.019***	-21.269***
	(0.568)	(4.594)	(0.547)	(6.902)	(6.890)
ρ	0.279**				
	(0.109)				
sigma2_e	0.011***				
	(0.000)				
N	3,036	3,036	3,036	3,036	3,036
*R* ^2^	0.151	0.151	0.151	0.151	0.151

Because the basic results of the SDM cannot directly reflect the direct effect and spatial spillover effect of each factor on carbon emission intensity, in order to further analyze, this paper divides the effects of each factor on carbon emission intensity into direct effect, indirect effect, and total effect according to Lesage and Pace’s [55] study. The results are shown in Table 6 (3)—(5). The coefficient of the linear term and the coefficient of the square term of the collaborative agglomeration of manufacturing and producer services are significant at the level of 1% in the direct effect, indirect effect, and total effect, and the coefficient of the linear term is positive, and the coefficient of the square term is negative. It indicates that the collaborative agglomeration of manufacturing and production service industries has a significant inverted U-shaped effect on the local and surrounding areas’ carbon emission intensity. From the perspective of coefficient values, the indirect effect of collaborative agglomeration between manufacturing and producer services is stronger than the direct effect, indicating that there is a substantial spatial spillover effect of the impact of collaborative agglomeration between manufacturing and producer services on carbon emission intensity. This verifies hypothesis 3. The industrial correlation between neighboring regions in China has gradually strengthened, especially in recent years. The application of various innovative technologies can promote cooperation between regions. The knowledge spillover generated by the collaborative agglomeration of manufacturing and producer services can also promote technological innovation in neighboring regions through spatial correlation, thus affecting carbon emission intensity. At the same time, as mentioned above, there is a "demonstration effect" among various regions in China, in which the coordinated development of industries in one region can drive the development of neighboring regions. Therefore, it is necessary to strengthen the joint regional development of manufacturing and producer services to give full play to their regional collaborative emission reduction effect and achieve the goal of reducing carbon emission intensity in the whole region.

For the control variables, among the direct effects, all control variables have significant effects on carbon emission intensity at the 1% level, except for environmental regulation. Only the spatial effects of energy intensity, urbanization rate, and human capital passed the significance test in the indirect effects. Among them, the coefficient of energy intensity is significantly positive in all three effects. Given that energy consumption is the main contributor to pollution emissions, it follows that an increase in energy consumption will result in a corresponding increase in pollution emissions within local and surrounding areas. It can also be attributed to regional economic competition and industrial correlation effects. The coefficient of the urbanization rate is significantly positive in the direct effect and significantly negative in the indirect effect; that is, the improvement of the urbanization rate in the region will enhance the carbon emission intensity in this region but will restrain the carbon emission intensity in the surrounding areas. Because of the acceleration of the urbanization process, the population is gathering in cities and cities, and the infrastructure is also increasing, which will produce many exhaust emissions. The coefficient of human capital is significantly positive in the direct effect and significantly negative in the indirect effect, indicating that human capital can significantly suppress the carbon emission intensity in the surrounding areas.

### 5.3 Robustness tests

The above analysis demonstrates that the collaborative agglomeration of manufacturing and producer services has a significant effect on carbon intensity in an inverted U-shaped curve. In order to further verify the credibility of this association, this paper conducts robustness tests using four different methods:

#### (1) Replace the dependent variable

According to the practice of Shao et al. [38], this paper replaced the dependent variable with per capita carbon emission(PCEI) to observe whether the conclusion was still valid. Column 1 of Table 7 shows the results of replacing the dependent variable with carbon emissions per capita. It can be seen that the coefficient of linear term the collaborative agglomeration of manufacturing and producer services is still significantly positive, and the coefficient of the square term is still significantly negative. It is consistent with the above conclusion, indicating that the result is robust.

**Table 7:** Regression results of robustness test.

Variables	(1)	(2)	(3)	(4)	(5)	(6)
	Replace the dependent variable	Eliminate some samples	Direct effects	Indirect effects	Total effects	GMM
L.CEI						0.311***
						(0.054)
COA	0.764**	0.202***	0.184***	4.758**	4.942**	0.560***
	(0.311)	(0.069)	(0.045)	(1.925)	(1.934)	(0.172)
COA^2^	-0.181***	-0.039***	-0.037***	-1.035***	-1.071***	-0.108***
	(0.067)	(0.014)	(0.009)	(0.400)	(0.402)	(0.033)
Control variable	yes	yes	yes	yes	yes	yes
N	3,036	3,036	3,036	3,036	3,036	2484
*R* ^2^	0.954	0.909	0.084	0.084	0.084	

#### (2) Eliminate some samples

In this paper, 1% is eliminated at both ends of the whole sample. Table 7 (2) shows the results obtained after removing part of the samples. It can be seen that the coefficients of linear term and square term of the collaborative agglomeration of manufacturing and producer services are both significant at the level of 1%, which once again proves that the collaborative agglomeration of manufacturing and producer services has an inverted U-shaped influence on carbon emission intensity. The robustness of the results is proved.

#### (3) Replace the spatial weight matrix

Since different spatial weight matrices may produce different results, this paper replaces the 0–1 spatial matrix with the spatial inverse distance matrix for robustness tests, such as model 6. The results obtained using the spatial inverse distance matrix are shown in columns (3)—(5) of Table 7. Among the direct effects, indirect effects, and total effects, the sign and significance of the regression coefficient of the collaborative agglomeration of manufacturing and producer services are consistent with those above, which further proves the robustness of the results.

#### (4) Endogeneity test

In order to solve the problem of reverse endogeneity between the collaborative agglomeration of manufacturing and producer services and carbon emission intensity, this paper introduced a one-stage lag of carbon emission intensity into the model (4) and used the GMM method for testing. The result shows that AR (2) is 0.866 greater than 0.1, indicating that the null hypothesis cannot be rejected, that is, *ε*_*it*_ does not exist autocorrelation. The Hansen test result of 0.297 is more extensive than 0.1, indicating that the null hypothesis cannot be rejected. It means that all the instrumental variables are exogenous, and the instrumental variables are valid. The results of the relevant estimation coefficients are shown in column (6) of Table 7. The coefficient of carbon emission intensity lagging one stage is significantly positive, and the linear term and square term coefficients of the collaborative agglomeration of manufacturing and producer services are both significant. The results are consistent with those mentioned above, which proves the robustness of the results.

### 5.4 Heterogeneity analysis

In order to investigate the difference in the impact of collaborative agglomeration of manufacturing and producer services on carbon emission intensity, this paper analyzed it from two aspects: industry heterogeneity and regional heterogeneity.

This paper subdivides the productive service industry into the high-end productive service industry and the low-end productive service industry. It then analyzes the impact of collaborative agglomeration between the high-end productive service industry and the manufacturing industry on carbon emission intensity, as well as the impact of collaborative agglomeration between the low-end productive service industry and the manufacturing industry on carbon emission intensity, respectively. Empirical results are shown in columns (1) and (2) of Table 8. The index of collaborative agglomeration of high-end producer services and manufacturing industries is positive in the linear term and negative in the square term, and both are significant at the 5% level. However, the coefficients of both linear term and square term of the index of collaborative agglomeration of low-end producer services and manufacturing industries are insignificant. The findings indicate that the collaborative agglomeration of manufacturing and high-end producer services is more effective in reducing carbon emission intensity compared to low-end producer services. It may be attributed to the fact that low-end producer services (including Transportation, storage, post and telecommunications industry, Wholesale, and retail trade industry, Water, environment and public facilities management industry, and Leasing and business services industry) are less knowledge-intensive and can generate limited knowledge spillover effects [16]. Additionally, the collaborative agglomeration of low-end producer services and manufacturing industries also have limited potential in creating new technologies to improve energy efficiency. The high-end producer services include Information transmission, computer services and software industry, Scientific research, technical services industry, and Financial industry. These industries possess a significant demand for knowledge and technology, and they also exhibit a strong spillover effect in terms of knowledge and technology. And this spillover effect can provide advantages for industrial collaborative innovation and reduce environmental pollution.

**Table 8:** Regression results of heterogeneity.

Variables	(1)	(2)	(3)	(4)
	High-end	Low-end	East	Central and West
COA	0.088**	0.016	0.388**	0.145*
	(0.044)	(0.130)	(0.167)	(0.088)
COA^2^	-0.022**	-0.021	-0.079***	-0.027
	(0.011)	(0.026)	(0.029)	(0.021)
Control variable	yes	yes	yes	yes
_cons	2.405***	2.478***	2.508***	2.239***
	(0.214)	(0.266)	(0.495)	(0.233)
N	3036.000	3036.000	1100.000	1936.000
*R* ^2^	0.902	0.903	0.896	0.909

**Fig 6:**
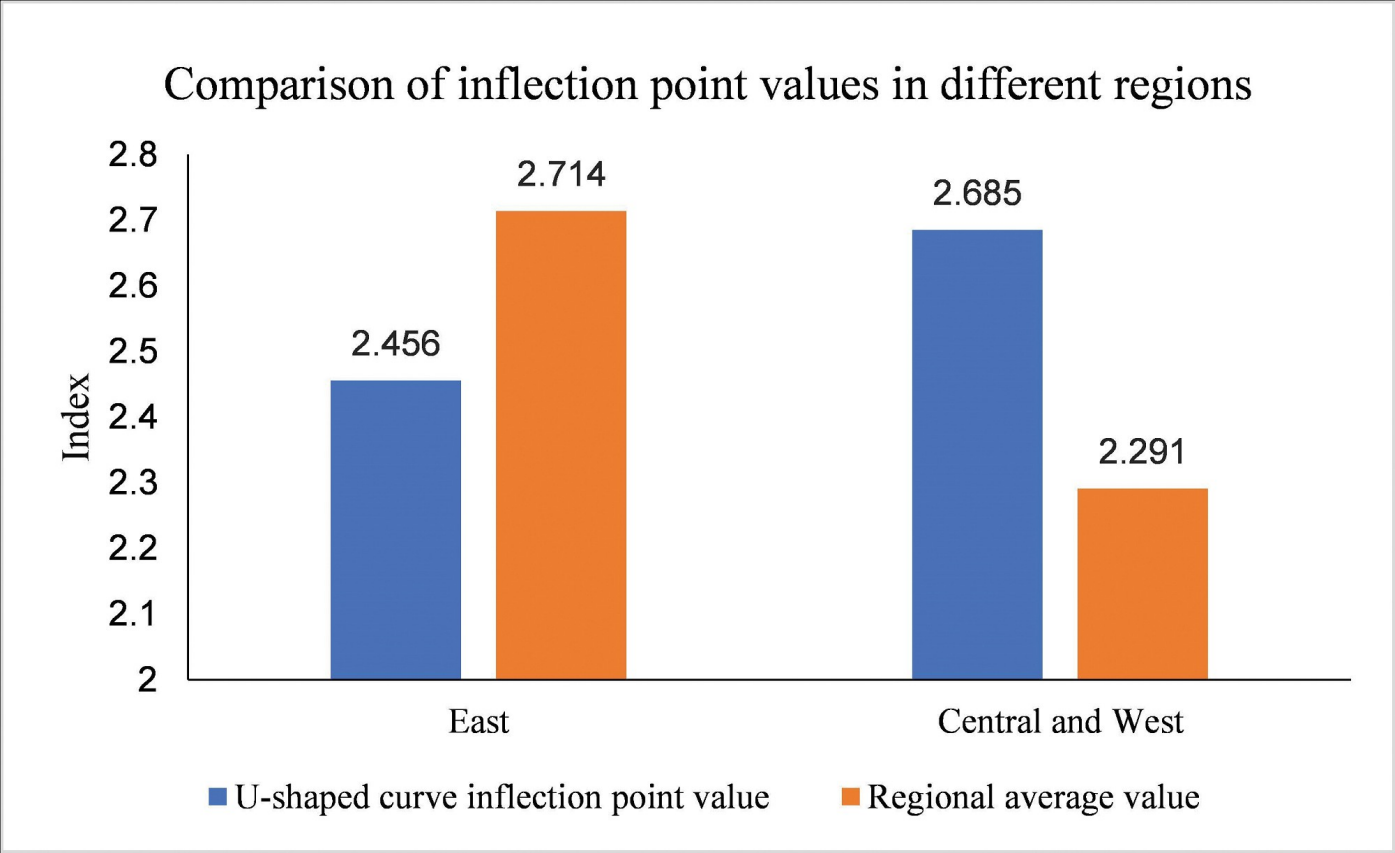
Comparison of inflection point values between eastern and midwestern regions.

The results of regional heterogeneity are shown in columns (3) and (4) of Table 8. The collaborative agglomeration of manufacturing and producer services in the eastern region significantly influences carbon emission intensity in an inverted U-shaped curve. However, the collaborative agglomeration of manufacturing and producer services in the central and western regions has no apparent influence on carbon emission intensity. It is possible that the relatively high level of economic development in the eastern region, along with the strong collaborative agglomeration between manufacturing and productive service industries, contributes to this phenomenon. Therefore, the exchange, integration, and collaborative innovation between the two major industries in the eastern region can play a better role. Regarding the inflection point of the collaborative agglomeration of manufacturing and producer services to curb carbon emission intensity, the inflection point of the eastern region is 2.456, while the inflection point of the central and western regions is 2.685. When the index of the collaborative agglomeration of manufacturing and producer services industries exceeds the inflection point, the carbon emission intensity can be suppressed. As shown in Fig 6. At present, the average collaborative agglomeration index in the eastern region is 2.714, which has crossed the inflection point and played a role in curbing carbon emission intensity. However, the average value of the collaborative agglomeration index in the central and western regions is 2.291, which has not to reach the inflection point of suppressing carbon emission intensity (Fig 6). It indicates that the level of collaborative agglomeration between manufacturing and producer services in the eastern region of China predominantly concentrated on the right side of the inverted U-shaped curve, which can give full play to the effectively mitigate carbon emission intensity. However, the level of collaborative agglomeration of manufacturing and producer services in China’s central and western regions is mainly distributed on the left side of the inverted U-shaped curve, which has not reached the extent of suppressing carbon emission intensity. Therefore, it is necessary to vigorously promote the agglomeration of manufacturing and producer service industries in the central and western regions of China so that they can cross the inflection point as soon as possible and play a carbon emission reduction effect.

## 6. Conclusions and recommendations

### 6.1 Conclusions

Based on the panel data from 276 cities in China, this paper studied the impact of the collaborative agglomeration between manufacturing and producer services on carbon emission intensity by establishing a fixed-effect model, intermediary model, and SDM, and draw the following conclusions:

First, in general, there is a significant inverse U-shaped relationship between the collaborative agglomeration of manufacturing and producer services and carbon emission intensity. The collaborative agglomeration of manufacturing and producer services will promote carbon emission intensity at the initial stage, and will suppress carbon emission intensity when the collaborative agglomeration index crosses the inflection point and reaches the maturity stage of agglomeration.

Second, technological progress plays an intermediary role in the process of industrial co-agglomeration affecting carbon emission intensity. The collaborative agglomeration of manufacturing and producer services can not only directly influence carbon emissions but also affect carbon emission intensity through technological progress. In addition, under the influence of technological progress, the inflection point value of the collaborative agglomeration of manufacturing and producer services to suppress carbon emission intensity becomes smaller, which can exert the inhibition effect on carbon emission intensity in advance.

Third, this paper selected data at the prefecture-level city level, allowing for a more detailed examination from a spatial perspective. From the spatial perspective, there is a significant spatial correlation between carbon emission intensity in China, and the carbon emission intensity shows a significant "high-high" and "low-low" agglomeration trend. According to the results of the SDM, there is a significant spatial spillover effect of the collaborative agglomeration of manufacturing and producer service industries on carbon emission intensity. From the spatial perspective, the collaborative agglomeration of manufacturing and producer service industries still has a significant inverted U-shaped curve effect on carbon emission intensity. The collaborative agglomeration of manufacturing and production service industries can first promote and then suppress the carbon emission intensity of neighboring regions through the spatial spillover effect. Furthermore, the spatial spillover effect of manufacturing and production service collaborative agglomeration on carbon emission intensity in neighboring areas is more evident than the direct effect on local carbon emission intensity.

Fourth, from the perspective of industry heterogeneity, the collaborative agglomeration of manufacturing and high-end producer services can play a more critical role in curbing carbon emission intensity than low-end producer services. From the perspective of regional heterogeneity, in the eastern region, the collaborative agglomeration of manufacturing and producer service industries can significantly have an inverted U-shaped impact on carbon emission intensity. However, in the central and western regions, the collaborative agglomeration of manufacturing and production service industries does not significantly impact carbon emission intensity. In addition, the level of collaborative agglomeration of manufacturing and production service industries in the eastern region has crossed the inflection point to play the role of carbon emission reduction, mainly distributed on the right side of the inverted U-shape. In contrast, the degree of collaborative agglomeration between the manufacturing and production service industries in the central and western regions remains situated on the left side of the inverted U-shaped curve, indicating that it has not yet had a significant impact on reducing carbon emission intensity.

### 6.2 Recommendations

Based on the above conclusions, and taking into account the actual situation in China, this paper puts forward the following recommendations:

First, in general, it is necessary to promote urban industrial agglomeration, especially to continue to vigorously promote the collaborative agglomeration of manufacturing and producer service industries. It is necessary to enhance the level of collaborative agglomeration between manufacturing and productive service industries, which can suppress carbon emission intensity through direct and indirect means to improve environmental quality. Moreover, it is necessary to optimize the internal structure of the producer service industry and strengthen the collaborative agglomeration of the high-end productive service industry and manufacturing industry. The communication and integration of the high-end productive service industry and manufacturing industry can reduce the manufacturing industry’s cost, improve the manufacturing industry’s efficiency and promote the reform and development of the manufacturing industry. Thus, it is beneficial to reduce carbon emission intensity and urban environmental pollution.

Second, relevant enterprises should fully utilize the intermediary role played by technological progress. As the supplier of technological innovation, the enterprise sector needs to be the focus of protection and support by policy. It is necessary to shift the path of technological progress from primarily relying on technology introduction to one primarily driven by independent research and development so as to grasp the intensity of industrial agglomeration more proactively. Although the scale effect brought by industrial collaborative agglomeration is important to the development of the industry, more attention should be paid to how to play the external effect of industrial collaborative agglomeration to promote the engine role of technological progress in the development of low-carbon economic transformation. In the future, it is still necessary to focus on the R&D and popularization of green technology in the process of industrial collaborative agglomeration to actively promote the combination of industry, academia, and research. It is also necessary to encourage innovation in the enterprise sector and foster a tremendous enthusiasm for innovation in the enterprise sector.

Third, each region should strengthen its industrial linkages with neighboring regions and pay attention to the spatial spillover effect of industrial collaborative agglomeration on carbon emission intensity in communication and cooperation with other regions. Regions should not only pay attention to the development level of their own manufacturing and productive service collaborative agglomeration and carbon emission intensity but also pay attention to the development level of the manufacturing and productive service collaborative agglomeration and carbon emission intensity of neighboring regions. It is imperative to enhance the communication and collaboration among regional industries. The government should support the synergistic linkage of inter-regional industries so that they can play a regional synergy effect, achieve the goal of reducing carbon emission intensity in the whole region, and achieve sustainable development.

Fourth, at the regional level, it is imperative to promote industrial collaborative agglomeration based on local conditions and achieve differentiated development. This is because there are variations in the collaborative agglomeration of manufacturing and producer service industries across different areas. In addition, compared to the central and western regions, the collaborative agglomeration of manufacturing and producer service industries in the eastern region exhibits a more pronounced inhibitory impact on carbon emission intensity. Therefore, the government needs to assist in adjusting the pace of synergistic industrial agglomeration development in different regions: the eastern region should continue to take advantage of its location, industrial and economic advantages to strengthen the development of high-end productive service industries and improve its technology level. Promote the communication and integration of high-end productive service industries with manufacturing industries, and play the carbon emission reduction effect of collaborative agglomeration. The central and western regions should vigorously promote the synergistic clustering of industries. They can use their industrial bases to establish industrial clusters and integrate resources through the mutual influence of knowledge communication, government support and technological progress. So that the collaborative agglomeration index of the central and western regions can cross the inflection point as soon as possible to play a role in carbon emission reduction and realize the overall environmental improvement of China.

The limitations of this study are as follows: firstly, due to the availability of data, this paper does not include data from all cities in China. Secondly, besides the control variables selected in this paper, there may be some factors that have an impact on carbon emission intensity, which scholars can further study in the future. Finally, in the future, we can conduct research on heterogeneity by dividing the sample into resource-based cities and non-resource-based cities based on whether they are resource-based cities.

## Supporting information

S1 File (XLSX)
